# Global Biases in Ecology and Conservation Research: Insight From Pollinator Studies

**DOI:** 10.1111/ele.70050

**Published:** 2024-12-31

**Authors:** Oksana Skaldina, James D. Blande

**Affiliations:** ^1^ Department of Environmental and Biological Sciences University of Eastern Finland Kuopio Finland; ^2^ Department of Biology University of Turku Turku Finland

**Keywords:** accessibility, biodiversity, conservation, environmental policy, funding, open science, pollinators

## Abstract

In the fields of ecology and conservation, taxonomic and geographic biases may compromise scientific progress. Using pollinator research as a case study, we evaluate four drivers of these biases and propose solutions to address (i) untested generalisations from highly studied taxa, (ii) information accessibility, (iii) scattered environmental regulations and (iv) restricted infrastructure and funding resources. Expanding the taxonomic, functional and geographic breadth of research and legislation, and involving scientists in policymaking, can generate greater equity, accessibility and impact of future science. Using search engines in different languages, Open Access (OA) publishing and promoting mutually beneficial collaborations between scientists from developed and developing countries, may help to overcome geographic biases in research and funding. We suggest reviewing potentially similar biases and their drivers in other branches of ecology and conservation and identifying further ways to achieve information balance in science.

## Graphical abstract

The viewpoint article discusses global biases and their drivers in ecological research such as untested generalisations from highly studied taxa, data accessibility, legislative and funding restrictions utilising a case study of pollinator research area. In the pollination ecology, most information is available for the restricted number of species (predominantly for bees) and for the restricted areas (Europe and North America). The article suggests potential solutions to mitigate the global biases in ecology and conservation research.

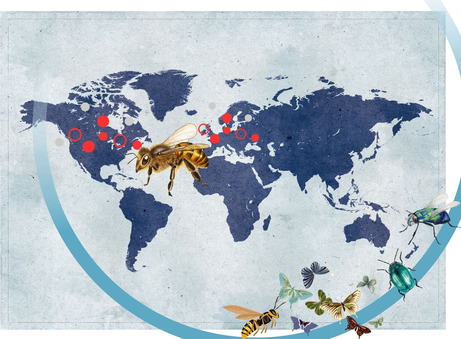

## 1 Problem Statement

**FIGURE 1:**
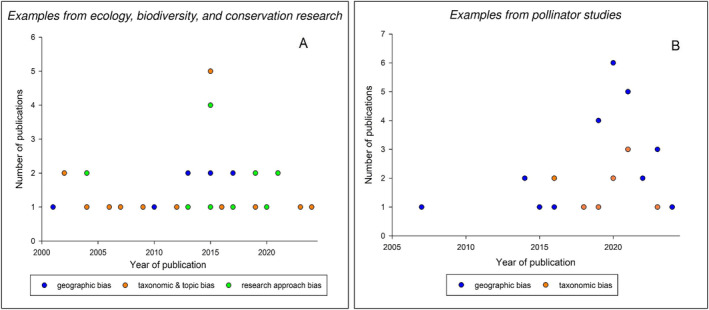
Scientific publications indicating various biases (geographic, taxonomic & topic and scientific approach biases) in ecology, biodiversity and conservation research published in the English language and extracted from the Web of Science for the years 2001–2024. Data points indicate the number of publications per year. (A) Research publications related to general ecology and conservation subjects including subject‐specific areas beyond pollinator studies. (B) Research publications indicating biases in pollinator studies. The associated datasets and metadata are available as Supporting Information.

Research biases compromise the quality of science. In ecology and conservation science, biases include (i) geographic bias—spatially scattered information (Trimble and van Aarde 2012), (ii) taxonomic & topic bias—dominance of charismatic taxa (Troudet et al. 2017) or historically popular topic(s) (Raerinne 2023) and (iii) scientific approach bias—hypothesis testing versus disconfirming evidence (Cassey et al. 2004; Leimu and Koricheva 2004) (Figure 1A). We suggest evaluating drivers of these biases and identify possible mitigation solutions using examples from pollinator research. We encourage ecologists from other research areas to review potentially similar area‐specific aspects.

## 2 Preface

**FIGURE 2:**
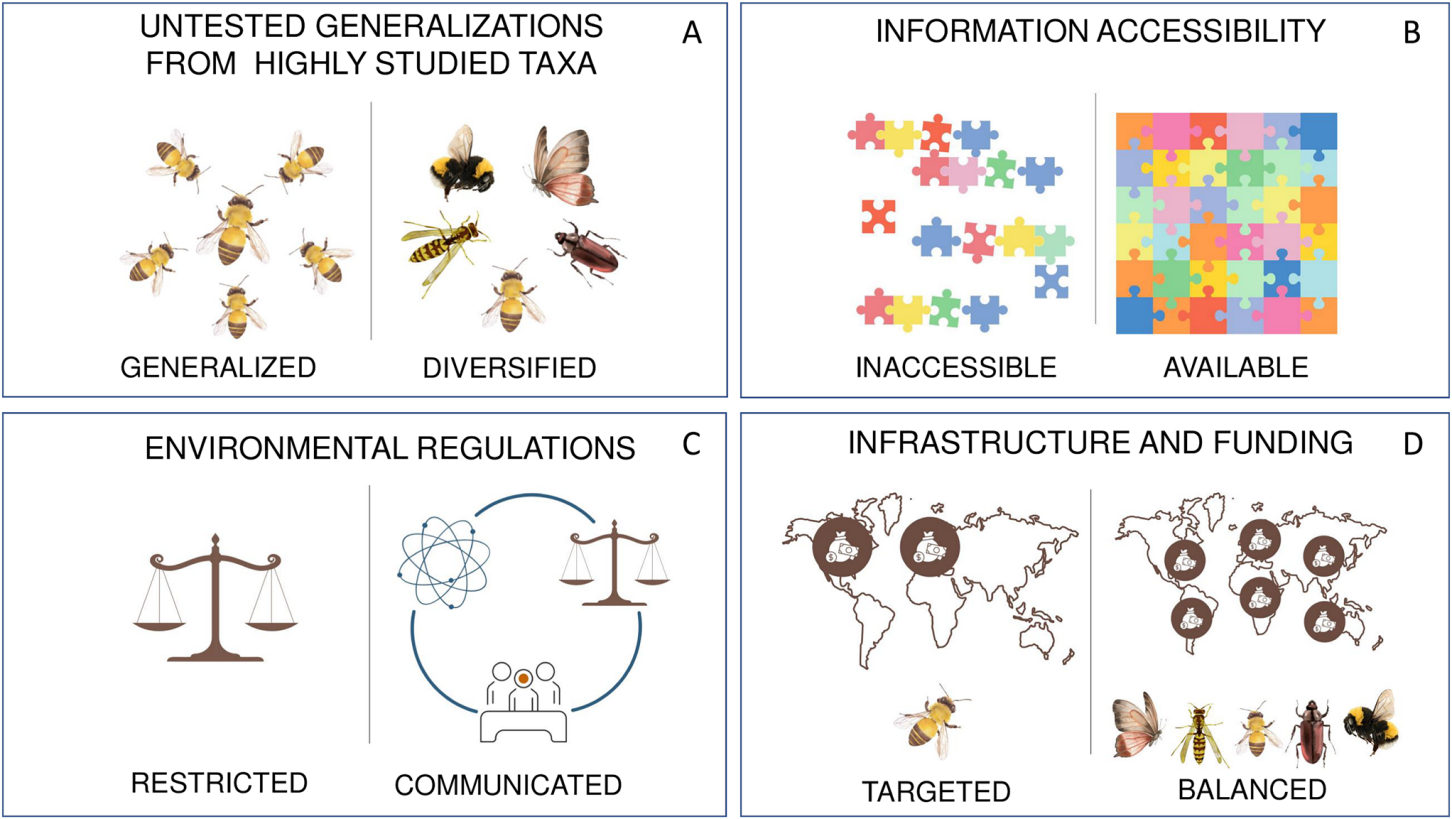
Four drivers of the global bias in ecology and conservation research illustrated with pollinator studies. (A) Generalising pollinators to ‘bees’ leads to bias in the pool of accessible information on other pollinating insects. (B) Using internationally accessible information published in English (indicated with orange colours of puzzles) facilitates a geographic‐bias gap, ignoring information available in other languages indicated with yellow, pink, red, blue and green colours; it also favours promotion of the ‘bee‐generalised’ pollinator concept. (C) The lack of systematic involvement of local communities and scientific experts in decision‐making leads to scattered environmental policies and promotes policies predominantly for charismatic taxa. (D) Funding programmes supporting restricted geographic areas and certain charismatic pollinator taxa deepen existing gaps.

Biases in ecology and conservation research have not decreased over time (Caldwell et al. 2024). There are documented global biases in pollinator studies, which manifest in research having a restricted geographic range and a narrow taxonomic focus with a lean towards species essential for agriculture (i.e., bees, and especially the western honey bee *Apis mellifera* L. 1758) (Figure 1B). To our knowledge, in pollinator studies, there are no articles published on the topic of scientific approach bias. We suggest that there are four interconnected drivers of these biases, which include untested generalisations from highly studied taxa, information accessibility, scattered environmental regulations and restricted infrastructure and funding resources (Figure 2).

## 3 Biased Geographic Scope in Research

Geographic bias is well documented in biodiversity conservation research, with approximately 75% of published studies focused on North America and Europe (Trimble and van Aarde 2012). Several studies in pollinator research have also documented a profound geographical bias towards North American and Western European territories and faunas (Brom, Underhill, and Winter 2022; Dicks et al. 2021; Orr et al. 2021; Porto et al. 2020; Silva et al. 2021). Geographic areas including Eastern Europe, Central and Eastern Asia, the Middle East, Northern and Central Africa, and Central and Southern America severely lack comprehensive information. Many of these regions have unique fauna, which is not comparable with other parts of the globe.

Asia is the richest continent in terms of honey bee diversity hosting eight indigenous *Apis* species ( *A. cerana* Fabricius, 1793; *A. florea* Fabricius, 1787; *A. andreniformis* F. Smith, 1858; *A. dorsata* Fabricius, 1793; *A. laboriosa* Smith, 1871; *A. nigrocincta* F. Smith, 1861; *A. nuluensis* Tingek, Koeniger & Koeniger, 1996; and *A. koschevnikovi* Enderlein, 1906) (Corlett 2011; Yang et al. 2011). Eastern Asia and specifically China has immense and vastly understudied bee diversity, being home to nearly half of the world's bumble bee diversity (125 species), including 22 that are endemic to the region (Teichroew et al. 2017; Huang and An 2018). Similarly, Mexico hosts nearly 10% of the world's butterfly (1800) and beetle (35,500) species, many of which are important pollinators (Promote Pollinators 2023). Countries such as Brazil, Indonesia, Argentina, Malaysia, Spain, Cote d'Ivore, Ukraine and Chile were reported among the greatest net exporters of pollinated crops (Murphy et al. 2022). Although recent efforts have been undertaken to increase knowledge on local pollinators for some of these countries (Muschett and Fontúrbel 2022; Nava‐Bolaños, Osorio‐Olvera, and Soberón 2022; Sabatino, Rovere, and Meli 2021), there remains a general lack of data.

There are studies that have sought to find ways to overcome this geographic bias. Strategies include using global databases, such as the Global Biodiversity Research Facility (GBIF 2024) or PREDICTS database (the project analysing ecological studies from around the world operated by the Natural History Museum, London) (Millard et al. 2021). Another approach is engaging international experts into direct discussions, as has been shown by Dicks et al. (2021) with the Delphi method (structured communication technique) or creation of the Database of Pollinator Interactions (DoPI) (Balfour et al. 2022). However, it still appears that the most biodiverse regions of the globe experience the greatest deficit in knowledge.

## 4 Biased Taxonomic Scope in Research

Taxonomic bias in biodiversity research is driven by societal preferences towards charismatic species (Small 2011; Sumner, Law, and Cini 2018). Generally, studies of plants and charismatic vertebrates are over‐represented (Troudet et al. 2017). In pollinator studies, there is a clear bias towards bees (Apoidea) (Millard, Freeman, and Newbold 2020). Media and journalistic misinterpretations have resulted in disproportionate attention to western honey bees ( *A. mellifera* ) (Ollerton 2017). Within Australian mainstream media publications over a 9‐year period, it was called ‘*the most important*’ or even ‘*only insect pollinator relevant to Australia*’ (Smith and Saunders 2016). Meanwhile only 15% of publications mentioned native bee species while 17% of papers were focused on non‐bee pollinators (Smith and Saunders 2016). A comprehensive review of 40‐years of pollination ecology studies in China, states that most research was focused on bee pollination despite the importance of studying pollinator diversity more broadly beyond a limited number of specific taxa (Ren et al. 2018).

## 5 Four Drivers of Geographic and Taxonomic Biases: Examples From Pollinator Studies

### 5.1 First Driver: Untested Generalisations From Highly Studied Taxa

Ecological concepts can be inefficiently simplified via generalisation. For example, generalising pollinators to bees has a reasonable biological root, as bees are the only group of insects that mostly rely on floral resources during immature stages (Winfree, Bartomeus, and Cariveau 2011) with only rare exceptions such as necrophagous bees (see Roubik 1982). However, such an approach has also led to less research being conducted on non‐bee pollinators resulting in a taxonomic bias. Major groups of pollinating insects aside from bees (Apoidea) include flower‐visiting flies (Diptera), wasps (Hymenoptera), butterflies and moths (Lepidoptera) and beetles (Coleoptera) (Ollerton 2017). Of these flower visitors, the butterflies and moths are the most taxonomically diverse group (with over 140,000 species that visit flowers) (Ollerton 2017). However, their ecological role as pollinators remains underestimated (Hahn and Brühl 2016). Aculeate wasps have a reputation for being pests (Sumner, Law, and Cini 2018), however they pollinate at least 960 plant species with 164 species being obligate wasp pollinated flowers (Brock, Cini, and Sumner 2021). Among the diverse range of pollinators, nocturnal species, especially flies and beetles, have been largely overlooked in research (Macgregor and Scott‐Brown 2020). Overall, non‐bee (non‐Apoidea) insect pollinators perform from 25% to 50% of the total number of flower visits, although these numbers are not proportionally represented in the scientific literature (Rader, Bartomeus, and Garibaldi 2016) and reports (Halvorson et al. 2021).

#### 5.1.1 Solutions

To achieve information balance in pollinator research, we propose that researchers include data on ‘by‐catch’ of non‐target insects; if taxonomic expertise for species‐level identification is unavailable, then reporting at broader taxonomic levels can nonetheless contribute to our understanding of the potential role of these understudied taxa. Increasingly, computer vision tools can be used to identify taxa from many orders (Høye et al. 2021). Journals can encourage submissions addressing taxa that are not only charismatic (e.g., bees) but also historically understudied (e.g., flies, wasps and beetles), while researchers can diversify their scope to previously unconsidered taxa. Authors of reviews and meta‐analyses could also consider being more mindful in their keyword searches to retrieve data more broadly besides charismatic species known to their fields.

### 5.2 Second Driver: Information Accessibility

Data inaccessibility in ecology and conservation research may be due to language, publication type, funding restrictions related to Open Access (OA) or the lack of standardised procedures. As noted by Archer et al. (2014), a sizeable pool of data on pollinators might be simply inaccessible. English is the major academic language of research publications. However, Amano, González‐Varo, and Sutherland (2016) revealed that 35.6% of 75,513 scientific documents published in 2014 were not written in English. Publishing in English in OA journals may pose a financial burden and be time‐consuming to scientists from developing countries with a different native language (Ramírez‐Castañeda 2020; Steinhardt et al. 2023). Ignoring knowledge published in languages other than English in not internationally recognised OA journals can result in a bias in every ecological topic (Amano, González‐Varo, and Sutherland 2016). Thus, the biased geographic scope of current pollinator research may represent a lack of information freely accessible in English, more so than a lack of existing knowledge.

The largest international research databases in the English language are Web of Science, Scopus, Pub Med, Science Direct and Justor (Paperpile 2023). However, other databases providing more complete search results in different languages and study regions are under‐utilised. Some of those databases include SciELO (Spanish and Portuguese), China National Knowledge Infrastructure CNKI (Mandarin), eLibrary (Russian), ETIS (Estonian), African Journals Online (studies from Africa), TRDizin (Turkish) and J‐Stage (Japanese) among others. These databases and data searches in various languages can provide essential information on case studies.

#### 5.2.1 Solutions

Information searches could be conducted in different languages and in a broader range of national/international databases. Language barriers could be overcome using services, such as Google Translate and Chat GPT, whereby artificial intelligence (AI) could assist in achieving a more comprehensive topic coverage. Facilitated international collaboration and partnership in science via training, visits and joint international publications might ease the cost burden of OA publishing for researchers possessing restricted language/financial resources.

### 5.3 Third Driver: Scattered Environmental Regulations

In ecological topics including pollinator conservation, isolated decision‐making has been linked to low engagement of scientists in environmental law, a lack of collective decision‐making and a narrow (agriculture‐oriented) focus of legislation (Hall and Steiner 2019; Hipólito et al. 2021). In the case of pollinators, precautionary conservation often appears ‘*too little and too late*’ (Drivdal and van der Sluijs 2021). Disconnection between science, policies and infrastructure produces an even more pronounced gap for developing countries (Martins 2021). Indeed, policymakers in developing countries often focus on species essential to agriculture (and often exclusively on honey bees), likely encouraging taxonomically and ecologically biased research (Hipólito et al. 2021).

The integration of science and law needs to overcome challenges such as different time frames and standards of proof, ethical obligations and the multijurisdictional (federal, provincial and indigenous) nature of environmental law (Hipólito et al. 2021; Moore et al. 2018). In various regions of the globe, there is still a low level of scientist participation in the development of environmental policies (Hipólito et al. 2021; Gemmil‐Herren et al. 2021; Ratamäki et al. 2015).

#### 5.3.1 Solutions

Scientists should be systematically involved in the decision‐making process, and thus graduate programmes should train future generations on how to communicate their science effectively with policymakers. Open platforms such as the Local Communities and Indigenous Peoples Platform (LCIPP) can be more broadly used. Legislation acts, such as the EU Nature Restoration Law, focusing on diversity and geographically local needs, might serve as templates for emerging policies.

### 5.4 Fourth Driver: Restricted Infrastructure and Funding

Many regions of the globe have poor research infrastructure and deficiencies in funding to perform comprehensive research. While reviewing the funding records related to pollination services, Porto et al. (2020) found that since 1992 around 95% of funded projects were affiliated to developed countries (primarily the USA and UK). This global imbalance further contributes to the geographic bias whereby most recent advanced studies were performed in the ‘Western world’. According to predictions by Murphy et al. (2022), developed economies will suffer the greatest economic loss imposed by pollinator decline as they depend on the pollination ecosystem services in developing countries. Projects focused on honey bees have also received more financial support compared to other pollinators (Small 2011).

#### 5.4.1 Solutions

Several integrated initiatives might be used to address this driver. These include the widespread use of global research infrastructures, such as the GBIF, PANGAEA (the data publisher for Earth & Environmental Science), open data hubs Dryad and Zenodo, via digitalised long‐term collection protocols, and by better organising local infrastructures (Bartomeus et al. 2019; Bartomeus and Dicks 2019). International funding of partnership programmes targeting both developed and developing countries, and joint proposals for creating solutions for common ecological targets, might help to achieve more balanced global knowledge and practice.

## 6 Conclusions

Future research should expand the breadth of study beyond the few charismatic species to reduce the problem of taxonomic bias. Public recognition of the biological and economic importance of these species could increase the interest and funding support to study understudied taxa in geographically underrepresented areas. Scientists and scientific institutions with sufficient funding and infrastructural resources can use other information than that available as OA in the English language and collaborate more with local specialists in the globally underrepresented areas. Policymakers need to account for broader needs and consolidate activities together with scientists and local communities. An increase in global funding and partnership programmes might help to tackle the problem of numerous biases in ecology, which could be made possible through intergovernmental, non‐profit and private fundraising efforts. While here we outline the bias in pollination science as a case study, we expect many globally important environmental and ecological questions to contain similar internal biases that urgently need to be reviewed and addressed.

## Author Contributions

O.S. developed the article concept. O.S. and J.D.B. wrote the manuscript.

### Peer Review

The peer review history for this article is available at https://www.webofscience.com/api/gateway/wos/peer‐review/10.1111/ele.70050.

## Supporting information


Data S1.

## Data Availability

Related data will be published at Zenodo repository: https://doi.org/10.5281/zenodo.14379344.
