# Supplementary material for: Global Biases in Ecology and Conservation Research: Insight From Pollinator Studies
Source: Ecol Lett. 2024 Dec 31;28(1):e70050. doi: 10.1111/ele.70050 (PMC11687349; doi:10.1111/ele.70050)
Supplement: Supplementary file 1 — Data S1. [file ELE-28-0-s001.zip › ele70050-sup-0002-SupinfoS2.docx]

**Global biases in ecology and conservation research:**

**insight from pollinator studies**

Oksana Skaldina1,2* and James D. Blande1

1 – Department of Environmental and Biological Sciences, University of Eastern Finland

2 – Department of Biology, University of Turku

*– corresponding author

Running title: Global research biases: pollinator ecology

**Metadata for the Supplement 1**

Supplementary information contains two data sets related to the **Figure 1A** and **Figure 1B**.

The data set “General biases” associated with **Fig. 1A** represents selected scientific publications in ecology, biodiversity, and conservation research published in the English language in 2001-2024 years. The table includes categories such as **No** (order number), **Reference** (complete reference of the selected paper), **Publication year** (year of publishing), **Research area** (specific field of ecology), and an **indication of the biases** such as geographic bias, topical bias, scientific research approach bias.

The data set “Biases in pollinator studies” associated with **Fig. 1B** represents selected scientific publications related to pollinator studies published in the English language from 2007-2024 that have indicated geographic and taxonomic biases. The table includes categories such as **No** (order number), **Reference** (complete reference of the selected paper), **Publication year** (year of publishing), **Region** (a geographic area on which is the current research focuses), **Pollinator group** (taxonomic group of pollinators which is in the focus of study), **Bias** (indication of geographic or taxonomic biases).
